# Sand-based therapy in pediatrics: a narrative review of traditional and digital sand therapy

**DOI:** 10.3389/fpsyg.2026.1692537

**Published:** 2026-02-10

**Authors:** Roi Jankelowitz, Brian Greeley, Maia Medland, Sima Zakani, John Jacob

**Affiliations:** Digital Lab, Faculty of Medicine, Department of Pediatrics and Orthopedics, Children’s Hospital, University of British Columbia, Vancouver, BC, Canada

**Keywords:** clinical outcome measures, digital sand therapy, narrative review, play therapy, sand-based therapy, sandtray outcome measures, sandtray therapy, pediatrics

## Abstract

**Introduction:**

Sand-based therapies, forms of play therapy, have been used to address emotional, behavioral, and psychosocial difficulties in children. Verbalizations, nonverbal cues, and depictions in the sand are used by therapists to assess client progress. In contrast, digital sand therapy uses digital apps or platforms to facilitate similar therapeutic processes. This narrative review examines the current state of evidence for sand-based therapies in pediatric populations with particular attention to the presence or absence of empirical research on digital sand therapy and their outcome measures.

**Methods:**

Inclusion criteria consisted of quantitative or mixed-methods sand-based therapy studies published between 2005 and 2025, involving pediatric populations and published in peer-reviewed journals. Searches were performed using PubMed, Google Scholar, PsycINFO, Scopus, Web of Science, and Embase, Perplexity AI, and the University of British Columbia library.

**Results:**

A total of 130 articles were identified. After screening, eight articles satisfied the inclusion criteria; there were no studies included that used virtual or digital sand-based therapy. All traditional sand-based therapy studies reported significant improvements in experimental groups. However, methodological limitations were common, including small sample sizes, a lack of between group comparisons, and an overall lack of objective outcome measures.

**Discussion:**

While traditional sand-based therapy studies suggest its potential effectiveness, marked limitations constrain the interpretations of these findings. Despite the promise and strengths of digital alternatives to traditional sand therapy, no empirical studies to date have examined their use in pediatric populations. While digital sandplay and sandtray platforms and applications exist, their clinical effectiveness has not yet been empirically studied.

**Conclusion:**

This review highlights a need for digital sand-based therapy in pediatric populations as well as standardized, objective measures for session analysis. Future research should explore how digital platforms can be used to enable objective sandtray analysis in pediatric populations.

## Introduction

Psychotherapy, also known as talk therapy, helps patients identify and change negative thoughts, emotions, and behaviors through conversation (Zablotsky and Ng, 2023). While psychotherapy can be effective in adults, it has been less effective in pediatric populations, as children lack the necessary cognitive and expressive capabilities needed to benefit from talk therapy (Benito Herce et al., 2024; Cuijpers et al., 2020; Garber et al., 2016; He et al., 2025). As such, it has been posited that children require age appropriate therapies (Garber et al., 2016).

Play therapy, a form of child therapy, aims to treat emotional, behavioral (Bratton et al., 2005), and psychosocial (Koukourikos et al., 2021) difficulties through play. Unlike psychotherapy, play therapy is tailored to children as it does not exclusively rely on verbal communication. Instead, play therapists use play as a means of communication and to observe behavior instead of verbal expressions. Subdisciplines of play therapy are sand-based therapies (e.g., sandtray and sandplay). In sandtray therapy, the therapist actively guides the play, whereas in sandplay therapy, the therapist’s role is passive and does not interfere with, or direct a person during a session (Koukourikos et al., 2021; Dobretsova and Wiese, 2019). Sand-based therapies highlight the potential of play-based methods as alternatives to psychotherapy for children.

Traditional sand-based therapies include a sandtray and a collection of miniatures (Chauhan et al., 2024). The sand can be wet or dry, and the miniatures typically include people, objects, and commonly feared creatures (Homeyer and Sweeney, 2022). Depending on the type of sand therapy, the therapist assesses patient progress by evaluating verbal and non-verbal cues as well as the completed sandtray. However, this analysis is highly subjective, contributing to limited empirical research on the subject. Digital sand-based therapies could be a promising alternative to traditional sand therapy. Like telehealth, an advantage of digital sand therapy could be that it is more inclusive, especially for children and adolescents living in rural, remote areas, increasing accessibility (Holliman and Foster, 2023). Additionally, digital sand therapy could offer the potential for objective measures of the sandtray, such as the speed of miniature selection and placement as well as the spatial arrangement of miniature placement, which could aid the therapist in analysis. In contrast, remote delivery of any therapy minimizes the therapist’s ability to observe non-verbal cues, which also play a role in analysis and the progress of the patient.

This review aims to understand the current state of both traditional (e.g., in-person sandplay and sandtray) and digital sand therapies and their outcomes. Our overall aim is to clarify the effectiveness of sand-based therapies and the types of outcome measures employed, to highlight both the opportunities and challenges within the field, and to understand whether digital sand-based therapy has been employed in pediatric populations and to determine its effectiveness.

## Methods

### Design

The purpose of this research was to synthesize findings and outcome measures in traditional (physical sandtray and miniatures) and digital (digital sandtray and miniatures) sand therapy studies that met an acceptable level of established study design criteria. To ensure consistency, we established a search strategy and quality assessment of each paper based on best practices (Gilson et al., 2025; Aguinis et al., 2023). Although this is a narrative review, we also followed standard Preferred Reporting Items for Systematic Reviews and Meta-Analyses (PRISMA) (Page et al., 2021).

### Search strategies

We conducted literature searches using standard search strategies under the two domains of digital and traditional sand therapies (play and tray). Search terms were derived from a variety of sources, such as keywords within abstracts, review articles, and Perplexity AI. We then used search platforms including: PubMed, Google Scholar, PsycINFO, Scopus, Web of Science, and Embase, Perplexity AI, and the University of British Columbia library. Searches were completed in June, July, and December of 2025. Search terms included: “digital sand therapy,” “digital play therapy,” “sand* therapy,” “online sand*,” “tele-play therapy,” “telemental health sand* therapy,” “digital Gestalt therapy,” “video game therapy,” “virtual sand* therapy,” technology sand* therapy.”

### Selection criteria

We reviewed articles that aligned with the goals of this review. Upon confirmation, we downloaded the relevant articles and coded them into a master spreadsheet. Eligible articles were high-quality, quantitative studies that examined either traditional or digital sand therapies in pediatric populations. Studies had to include a pediatric population as part of the age range, had to be published in a credible journal, and published within the last 20 years (2005–2025).

### Data extraction

Once articles were downloaded and coded into the master spreadsheet, we used ChatGPT (assessed June and July 2025, v4.0; December 2025 v5.2) to generate preliminary extractions of predefined information we wanted to extract from each of the articles (see Supplementary document for prompt). The extracted components included: year of publication, lead author, country of lead author, title, journal, article type, keywords at the bottom of abstract, novelty, questions/aims, sample size, participant age range and mean, participant sex, study type, description of intervention, groups, procedure, primary and secondary outcomes, results, gaps, and conclusion.

All ChatGPT-generated outputs were reviewed in detail by independent human reviewers (RJ, BG, or MM) to ensure accuracy, completeness, and appropriate interpretation of the source articles. Discrepancies were resolved by a third reviewer (BG). No AI-generated text was used in the review or its tables.

Following verification, all identified articles were screened using the predefined inclusion and exclusion criteria recorded in a standardized spreadsheet. Studies assessed as insufficient or of poor quality were flagged and confirmed by either RJ or BG. The remaining eligible articles underwent full-text review and summarization either by RJ or BG, in adherence to the established criteria.

### Quality assessment tool

To assess the overall quality the Mixed Methods Appraisal Tool 2018 version (Hong et al., 2018) was applied to all included studies. The tool is trichotomous, with the only answers being: “yes,” “no,” or “cannot tell.” Comments were included in sections where there was no definitive answer or in areas that required further explanation (see Table 1).

**Table 1:** Results of the MMAT quality assessment tool.

Author (year)	Study design type	Screening Q1	Screening Q2	Q1	Q2	Q3	Q4	Q5
Traditional sand therapies								
Rousseau et al. (2009)	RCT	Yes	Yes	2.1: Cannot tell Comment: Does not explain how randomization occurred.	2.2: Yes	2.3: Yes	2.4: No Comment: Parents and interviewers were blinded. Teachers were not.	2.5: No Comment: There was a 17% dropout rate in the pretest.
Yahaya et al. (2018)	RCT	Yes	Yes	2.1: Yes Comments: Randomization occurred within the classroom.	2.2: No Comment: Only one (3%) participant was 16 years old, the rest were 12 and 13.	2.3: Yes	2.4: No Comments: Blinding was not discussed at all.	2.5: Yes
Han et al. (2017)	Non-randomized trial	Yes	Cannot tell Comment: Did not perform between group comparisons.	3.1: Cannot tell Comments: Single site sample limits generalizability and there is a lack of standardized diagnostic criteria for externalizing behavior problems.	3.2: Cannot tell Comment: Measurements seem appropriate for answering research questions, but teachers were not blinded.	3.3: Yes	3.4: No Comment: Failed to consider key demographics such as SES, parental factors, and classroom variables.	3.5: Yes
Keivani and Alhosseini (2018)	RCT	Yes	Yes	2.1: Yes	2.2: Cannot tell Comments: Authors claim that the significant *p*-values (attention problems for the experimental group and thinking problems in the control and experimental group) were addressed, but the stated values were inconsistent with Table 2.	2.3: Yes	2.4: No Comment: No mention of blinding.	2.5: Yes
Matta and Ramos (2021)	RCT	Yes	Cannot tell Comments: Did not perform between group comparisons.	2.1: Cannot tell Comments: Randomization done at institutional level.	2.2: No Comment: Sex % in each group varies. The control group was 65% males, while the placebo group was 25%. Time at an institution also varied. The control group was 13.2 months, while the experimental group was 23.8 months.	2.3: No Comment: Failure to measure follow-up in the placebo and control groups.	2.4: No Comment: Does not discuss blinding.	2.5: No Comment: Eight participants (40%) did not complete the follow-up assessment (6 months later) in the experimental group.
Tan et al. (2021)	RCT	Yes	Yes	Yes	Yes Comment: Statistical trends (e.g., *p* = 0.078 and *p* = 0.087) were observed in primary outcome measures pre-intervention.	Yes	No	Yes
Li et al., (2022)	Mixed-Methods	Yes	Yes	Yes	Yes Comment: While true, sandplay sessions were spread out.	No	Yes	No Comment: 60 statistical comparisons completed. There was no correction for multiple comparisons.
Liu et al. (2023)	RCT	Yes	Yes	Yes	Yes	Yes Comment: 3 participants from experimental group were lost to follow-up, 4 participants from control group were lost to follow-up	No	No Comment: 43 participants completed 80% of the sessions, only 32 participants completed all sessions.

SIHEQ, Sandplay Image Healing Experience Questionnaire; SUS, System Usability Scale; WASI, Wechsler Abbreviated Scale of Intelligence.

## Results

### Articles excluded

**Figure 1:**
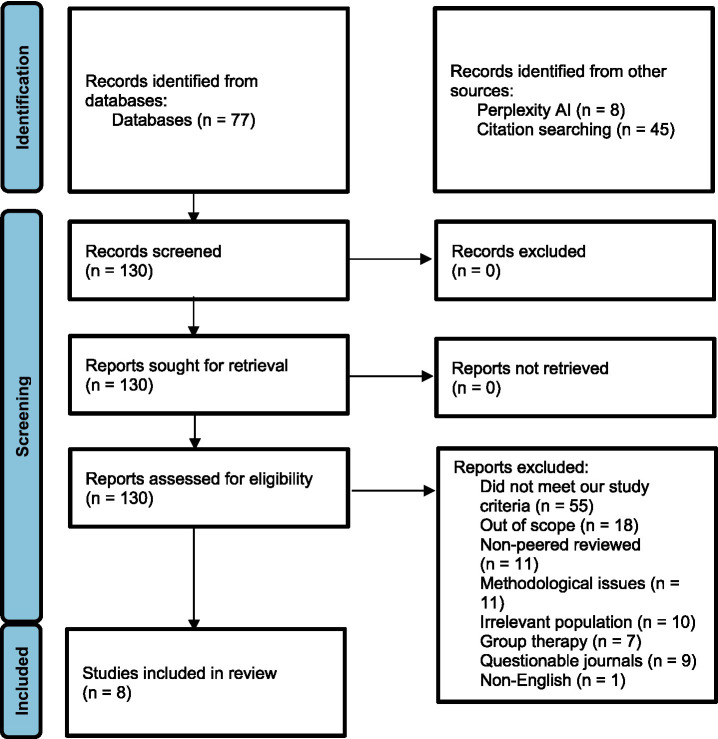
Preferred reporting items for systematic reviews and meta-analyses (PRISMA) flowchart.

One hundred and thirty articles were initially found and 122 articles were excluded. Of those excluded, 55 (42%) did not meet study criteria (e.g., case study, review, conceptual article), 18 (14%) were out of scope (e.g., focusing on unrelated therapies), 11 (8%) were from non-peer reviewed sources (e.g., popular science books, dissertations), and 11 (8%) were excluded due to methodological concerns (e.g., no control group, *n* < 10). Additionally, 10 (8%) involved irrelevant participants (e.g., all or some participants were adults), seven (5%) focused on group-based sand therapies, nine (7%) were published in journals not widely indexed in major databases, and one (1%) was not written in English. In total, eight articles were included (Figure 1). We found one digital sandplay article, but because it included adults and did not meet our criteria, it was not included in the results (see Discussion). The quality and a summary of the included articles can be found in Tables 1, 2, respectively.

**Table 2:** Results of the included studies.

Author (year)	Country	N	Age range (mean) and description	Number of females (%)	Setting	Intervention group (*n*) and dose	Control group (*n*)	Outcome measures	Results
Traditional sand therapies									
Rousseau et al. (2009)	Canada	105	4 to 6 year olds (5.3) from recently arrived immigrant and refugee families.	59 (56%)	Classroom	Sandplay (52). Sixty minute sessions, conducted every 2nd week over a span of 4 months.	Attended class as usual (53).	SDQ (parent and teacher reported) to measure emotional and behavioral problems.	The experimental group showed a reduction in SDQ teacher-reported total scores, specifically changes in emotional symptoms subscores (anxiety / depression) relative to the control group. All parent-reported SDQ scores were not significant.
Yahaya et al. (2018)	Brunei	32	Healthy 12 to 16 year olds (not reported).	16 (50%)	Not reported	Sandplay (16). Four weekly sessions were 45–60 min.	No intervention (16).	SEI (child reported) to measure 5 self-esteem variables (general, social, parental, academic, and lie).	General self-esteem increased in the experimental group relative to the control group. There were no significant differences in all other self-esteem variables.
Han et al. (2017)	Republic of Korea	20	4 to 5 year olds (not reported) with externalizing behavior problems.	4 (20%)	Childcare centers	Sandplay (10). 30 min sessions, conducted twice a week for 8 weeks.	No intervention (10).	Korean version of the PSBS (teacher reported) to measure aggression and PIPPS (teacher reported) to measure children’s peer interactions.	The experimental group had a reduction in physical and relational aggression and negative peer interaction, but not changes were seen in positive peer interactions. No changes observed in the control group. All changes in behavior were observed within groups. No between group comparisons were completed.
Keivani and Alhosseini (2018)	Iran	24	5 to 6 years old (not reported) with emotional behavioral problems.	12 (50%)	Not reported	Sandtray* (12). 30–40 min sessions, conducted twice a week, over 5 weeks.	No intervention (12).	CBCL (parent reported) to measure children’s emotional, behavioral, and social skills.	A reduction in anxiety / depression, social problems and aggression subscales of CBCL was found in the experimental group relative to the control group. There were no changes in isolation/depression, physical complaints, thinking or attention problems, or disobedience behavior.
Matta and Ramos (2021)	Brazil	60	6 to 10 years old (not reported) with internalizing and/or externalizing behavior problems living in shelters.	26 (43%)	Shelter	Sandplay (20). 45 min sessions, conducted once a week for 5 months.	a. Control (20) waitlisted for the duration of the study. b. Placebo (20) 45 min weekly sessions, playing with a sand box, a rake, and stones.	CBCL (unknown who reported) to measure children’s emotional, behavioral, and social skills.	In the experimental group, there was a reduction in externalizing (aggression and rule-breaking behavior) and internalizing behavior. The control group also reduced externalizing behaviors (aggression). The placebo group showed no behavioral changes. No between group comparisons were done.
Tan et al. (2021)	China	60	6 to 12 years old (9.3 for experimental group, 9.0 for control group) with leukemia or chronic kidney disease.	29 (48%)	Children’s hospital	Sandplay (30). Six sessions performed 1–2 times a week for 60–90 min.	Control group received regular nursing care (30).	CBCL (parent reported).	Group differences observed in the total score and the anxiety and attentional problems sub-scores. Those in the sandplay therapy group had greater reductions in total scores, anxious and attentional problems subscores relative to the control group.
Li et al. (2022)	China	40	Children and adolescents (age range not reported) with mild or moderate systemic lupus erythematosus. Mean age 15.6 for experimental group, 16.3 for control group.	37 (93%)	Hospital	Sandplay (20). Standard drug treatment with three sessions of sandplay each lasting 50 min performed at week 0, 2, and 4 after diagnosis.	Control group received standard drug treatment (20).	CDI-S (self-report), SCARED (unknown who completed survey), PedsQL (unknown who completed survey) 4.0, and blood work.	Observed multiple group differences at various timepoints. CDI-S and total SCARED scores were lower for the sandplay group at weeks 4 and 12 compared to the control group. Social function and school function subscores of PedsOL 4.0 were higher for the sandplay therapy group relative to control group at weeks 4 and 12. C3 and C4 were higher at weeks 2, 4, and 12 for the sandplay group compared to the control group.
Liu et al. (2023)	China	52	Aged 3 to 6 (mean not reported; median 4.7). Clinically diagnosed with autism spectrum disorder.	22 (42%)	Special education school for children	Sandplay (26). Parent–child sandplay therapy with Applied Behavior Analysis-based program. Sandplay therapy consisted of 20 sessions each 45–60 min in length.	Control group received 20-h weekly Applied Behavior Analysis-based program (26).	ABC (parent-reported), SRS (parent-reported), PSI-SF.	Those in the sandplay group displayed lower ABC, SRS, and PSI-SF scores at week 20 and 32 relative to those in the control group.

ABC, Autism Behavior Checklist; AR, Augmented Reality; CBCL, Child Behavior Checklist; CDI-S, Children’s Depression Inventory; PSBS, Preschool Social Behavior Scale-Teacher Form; PedsQL, Pediatric Quality of Life Inventory; PIPPS, Penn Interactive Peer Play Scale; PSI-SF, Parenting Stress Index-Short Form; SCARED, Screen for Child Anxiety Related Emotional Disorders; SDQ, Strengths and Difficulties Questionnaire; SEI, Self-Esteem Inventory; SRS, Social Responsiveness Scale; YSR: Youth Self Report; WASI: Wechsler Abbreviated Scale of Intelligence. *denotes the use of sandtray therapy as opposed to sandplay therapy.

### Traditional sand therapies (play and tray)

Rousseau et al. (2009) integrated sandplay therapy into kindergarten classrooms located in an area with a high concentration of recently arrived immigrant and refugee families. One hundred and five children, aged 4 to 6 (56% female), participated. The experimental group (*n* = 52) engaged in 10, 60-min sandplay sessions every 2nd week for 4 months, whereas the control group (*n* = 53) continued with their regular classes. Sandplay sessions were led by three art therapists. The success of the sandplay sessions was assessed using the Strengths and Difficulties Questionnaire (SDQ), as reported by both the parents and teachers. The SDQ is a 25-item questionnaire assessing emotional and behavioral symptoms, that consists of five subscales: emotional symptoms, conduct problems, hyperactivity-inattention, peer relationships, and prosocial behavior (Goodman, 2001). Results revealed a significant reduction in the teacher-reported SDQ total scores, more specifically, limited to the emotional symptoms subscale (anxiety and depression) for the experimental group relative to the control group. All parent-reported SDQ scores were not significant between groups.

A study by Yahaya et al. (2018) involved 32 healthy child and adolescent participants aged 12 to 16 (50% female). Participants in the experimental group (*n* = 16) underwent 4 weekly sandplay therapy sessions, each lasting 45 to 60 min. Sessions followed sandplay guidelines (Homeyer and Sweeney, 2022). The control group (*n* = 16) received no intervention. Self-esteem was the primary outcome, measured by the Self-Esteem Inventory, which measures five self-esteem variables (general, social, parental, academic, and lie) (Coopersmith, 2012). Researchers found that the experimental group had an increase in general self-esteem relative to the control group. No other group differences were found.

A study by Han et al. (2017) and colleagues included 20 participants, aged 4 to 5 (20% female), with externalizing behavioral problems. The experimental group (*n* = 10) received 30-min sandplay therapy sessions, twice a week, for a total of 16 sessions, conducted by a registered play therapist. Sessions were conducted at each participant’s childcare center. Participants in the control group (*n* = 10) received no intervention. To measure externalizing behavioural problems, specifically aggression, the teacher-rated Korean version of the Preschool Social Behavior Scale was used, consisting of 19 items and two subscales for relational and physical aggression (Crick et al., 2011). Additionally, negative and positive peer interactions were assessed using the Penn Interactive Peer Play Scale (Fantuzzo et al., 1998), a 20-item teacher rating scale assessing children’s peer interactions, with eight and 12 items measuring positive and negative interactions, respectively. Results indicated a reduction in both physical and relational aggression within the experimental group but not within the control group. Sandplay therapy also reduced negative peer interaction but did not affect positive peer interactions. The researchers did not perform comparisons between groups.

A study by Keivani and Alhosseini (2018) consisted of 24 children aged 5 to 6 (50% female). All participants had emotional-behavioral problems according to the Child Behavior Checklist (CBCL), a 113-item parent-reported questionnaire that contains subscales for internalizing (e.g., anxiety/depression, isolation/depression, and thinking problems) and externalizing (e.g., disobedience, aggression, and social problems) behavioral problems (Achenbach and Ruffle, 2000). The experimental group (*n* = 12) received sandtray therapy sessions lasting 30 to 40 min, conducted twice a week for a total of 5 weeks, under the guidance of a therapist. The control group (*n* = 12) received no intervention. The CBCL was also used as an outcome measure, completed after the 5-week program. Those in the sandtray therapy group showed a reduction in anxiety/depression, aggression, and social problems relative to the control group. However, sandtray therapy had no effect on isolation/depression, physical complaints, thinking problems, attention problems, or disobedience behavior.

Matta and Ramos (2021) conducted a study involving 60 participants, aged 7 to 10 (43% female), who lived in shelter institutions. Participants had borderline or clinical scores of internalizing or externalizing behavior problems, according to the CBCL. The experimental group (*n* = 20) received 20 weekly, therapist guided sandplay sessions, while the placebo group (*n* = 20) received 20 weekly sessions of play time with sand, a rake, and stones and the control group (*n* = 20) was waitlisted. Sessions were 45 min in duration. The experimental group demonstrated reductions in both internalizing and externalizing behavioral problems, while the control group displayed a reduction in externalizing behavior problems. Follow-up analysis revealed that the experimental group had a reduction in both aggressive and rule-breaking behavior, whereas the control group showed a reduction in aggressive behavior. No follow-up was completed on the internalizing behaviors. The placebo group showed no changes. No between group comparisons were performed. In addition to the behavioral measures, researchers also analyzed the emergence of positive (e.g., celebration) and negative (e.g., threat/conflict) themes from the experimental groups’ sandtrays. The researchers reported that over the course of the sessions that there was an increase in positive themes and a decrease in negative themes, but the authors provided no details in how positive and negative themes were determined.

An RCT conducted by Tan et al. (2021) included 60 children aged between 6 and 12 years old (48% female) that were receiving long-term care at a children’s hospital for either leukemia or chronic kidney disease. Patients were randomized into one of two groups: sandplay administered by sandplay therapy or standard of care. Those randomized into sandplay therapy received six sessions of sandplay therapy performed one to two times per week, with each session lasting between 60 to 90 min. In contrast, those randomized into the control group received regular routine care and daily expressions of “consolation and encouragement” by staff. The primary outcome measure in the study was the parent-reported CBCL, completed pre- and post-intervention. Total CBCL change scores (i.e., difference between pre- and post-intervention) were different between the two groups, with greater reductions in CBCL total scores seen in the sandplay therapy group relative to the control group. Similarly, change scores for the anxious and attentional problems sections within the CBCL were also and showed greater reductions in the sandplay therapy group relative to the control group.

A mixed-methods study conducted by Li et al., (2022) recruited 40 children and adolescents (93% female) with mild or moderate systemic lupus erythematosus. Patients were randomly assigned to a control group or an experimental group. The control group received standard drug treatment, whereas the experimental group received sandplay therapy administered by a physician at 0, 2, and 4 weeks after initial diagnosis in addition to drug therapy. All patients completed the Children’s Depression Inventory (CDI-S), a self-reported questionnaire to assess depression in children (Achenbach and Ruffle, 2000), the Screen for Child Anxiety Related Emotional Disorders (SCARED) (Birmaher et al., 1997) to measure anxiety, the PedsQL 4.0 (Varni et al., 2001), to measure quality of life, and a blood test that assessed the condition of their lupus at weeks 0, 2, 4, and 12. In addition to a linear mixed model, group comparisons were assessed at each timepoint for each score and subscore with no adjustment for multiple comparisons. The experimental group displayed lower CDI-S and SCARED total scores compared to the control group in weeks 4 and 12. Social and school function subscores of the PedsQL 4.0 were found to be higher in the experimental group compared to the control group in weeks 4 and 12.

An RCT conducted by Liu et al. (2023) and colleagues, recruited 52 children and their caregivers between the ages of 3 and 6 (42% female) that had a clinical diagnosis of autism spectrum disorder. Participants were randomly assigned to be enrolled in either a 20-h weekly Applied Behavior Analysis-based program (ABA; control group), or the ABA program supplemented with a parent–child sandplay therapy intervention (experimental group). Those in the experimental group received 20 sessions of sandplay therapy, with each session lasting 45–60 min in duration. The researchers wanted to know whether the ABA program with parent–child sandplay affected behavior, social interaction, and parenting stress (primary outcomes). Liu et al. used the Autism Behavior Checklist (ABC) (Krug et al., 1988), a 57-item covering five categories of autistic behaviors, the Social Responsiveness Scale (SRS) (Constantino, 2013), which measures social aspects of autism, and the Parenting Stress Index-Short Form (PSI-SF) (Abidin et al., 2006), a 15-item survey that measures parent’s stress levels. Questionnaires were collected at baseline and two timepoints post-intervention (week 20 and 32). Group comparisons revealed that the experimental group (sandplay therapy with the ABA program) had lower ABC, SRS, and PSI-SF scores at 20 and 32 weeks relative to the ABA program group.

## Discussion

Despite a thorough search of the literature, we found no digital sand therapy studies with pediatric participants. However, eight traditional sand therapy studies with children and adolescents were included in our narrative review. Overall, there was some empirical evidence to suggest the effectiveness of sand therapies, however many of the articles that found an advantage of sand therapy lacked scientific rigor. Common among these studies were small sample sizes, a lack of between group comparisons and objective sand therapy specific outcome measures.

Many of the published sand therapy studies we identified lacked scientific rigor. We identified 130 sand therapy articles, however 31 (24%) articles were excluded for either having no control group or having small sample sizes (e.g., *n* < 10), were not from a peer-reviewed source, or were published in journals not widely indexed in major databases. The studies that met our inclusion criteria, however, also contained numerous methodological concerns. The quality assessment of the included studies revealed unclear randomization processes, large participant dropouts, and an overall lack of transparency (Table 1). We consistently found that all the included studies had small sample sizes (average *N* = 49; average *n* = 23), with all but three studies (Rousseau et al., 2009; Matta and Ramos, 2021; Tan et al., 2021) having fewer than 60 total participants. This is markedly lower than the average of 93 participants typically seen in RCTs involving cognitive behavioral therapy (Thoma et al., 2012) and much lower than the recommended 60 per group (*N* = 120) for RCTs in general (Teare et al., 2014). Small sample sizes increase the risk of Type I errors, where non-significant findings are considered significant (Columb and Atkinson, 2016). These findings highlight a need for more rigorous, large-scale, and peer-reviewed research in sand-based therapy studies.

Studies investigating the use of virtual or digital sand therapy are rare. While our search identified a single peer-reviewed digital sand therapy study, it was excluded due to the characteristics of the sample. Xiong et al. (2022) enrolled 20 participants with mild to moderate anxiety and examined the effects of a single 35 min session of augmented reality (AR) sandplay therapy delivered using a mobile app (*n* = 10) compared with traditional sandplay therapy (*n* = 10) on stress. Stress was measured using galvanic skin response (e.g., skin conductivity) and systolic blood pressure. The authors observed a reduction in skin conductivity in the AR group, however, both groups displayed reductions in systolic blood pressure relative to baseline. This study was excluded from our review because participants were aged between 15 to 30 years old and did not meet our pediatric inclusion criteria. Despite its exclusion, a limitation of this study warrants attention. Notably, no direct group comparisons were completed in the statistical analysis. The authors interpreted their findings as evidence that AR sandplay reduced stress, a conclusion that overstates the results. A significant difference found within one group but not the other does not constitute evidence of a between group difference (Makin and De Orban Xivry, 2019); such a conclusion requires a direct statistical comparison. At present, no digital sandplay therapy studies in pediatric populations exist, and it is unknown how digital sandplay therapy compares to traditional sand therapy in children. Future research should explore these questions and explicitly perform between group comparisons with appropriate connection for multiple comparisons.

While we did not identify any empirical studies examining digital sandplay in pediatric populations, digital sandplay therapy apps and platforms do exist. One platform identified was Online Sand Tray, a free web-based platform that provides several hundred digital miniatures that users can copy, flip, and resize. The platform includes a save feature that allows users to download an image of the sandtray at any point. In theory, these saved images could be used to analyze the sandtray characteristics, such as the spatial relationships between the miniatures, which may offer insight into therapeutic processes or outcomes. However, the Online Sand Tray platform has several limitations. First, no empirical studies have examined its use. Second, the saved images do not capture the order or speed of miniature selection, nor the progression of the sandtray across a session (e.g., miniatures selected early but removed later), all of which may be clinically relevant. Despite these limitations, the Online Sand Tray platform represents a promising open source initial step in the development of digital sandplay therapy.

We also found two commercial (i.e., pay to play) digital sandplay apps. The Virtual Standtray app allows users to manipulate a virtual sandtray by enabling them to “dig down to a liquid layer, build up the sand, and paint the sand” and includes “over 7,000 3D models to place, resize, rotate, levitate, and more.” However, the app costs USD 169.99 to access, in addition to in-app purchases, the cost of which is unknown. Importantly, the developer and seller of the Virtual Sandtray app, Chris Ewing and Jessica Stone have not published peer-reviewed empirical papers on the app or its use. Several of Jessica Stone’s books were identified in our search but were excluded as they constitute gray literature and have not been peer-reviewed. Simply Sand Play is another digital sandplay app and allows users to select from a library of 500 items, and “create water, snow, grass, or mounded sand, rotate, copy, duplicate, stack, bury, and delete objects.” The app costs USD 9.00 a month, or USD 90.00 per year, however, like the Virtual Sandtray app and Online Sand Tray, no empirical research has examined its use. Thus, while digital sandplay and sandtray apps and platforms exist, their use has not been empirically studied and peer-reviewed. Future research can address this gap by systematically evaluating the clinical feasibility, usability, and therapeutic outcomes associated with digital sandplay interventions, particularly within pediatric populations.

Our review also revealed an overall lack of outcomes directly related to sand therapy. While clinical outcome measures are important and should be the primary outcome, patient behaviors during sessions, such as miniature selection and placement, non-verbal cues, and verbalizations, can also used for analyzing treatment effectiveness (Homeyer and Sweeney, 2022). Some sand therapists often analyze images or videos of the session to identify overarching themes. However, only one of the included studies used sandtray-specific outcome measures (Matta and Ramos, 2021). In their study, Matta and Ramos (2021) categorized trays into positive or negative themes. While theme analysis is highly subjective as it relies exclusively on the therapist’s interpretations, a major advantage of virtual sandplay is its potential to quantify sandplay related behavior (e.g., miniature selection and placement [in relation to other miniatures]). Other fields such as cognitive training have already established objective analytical approaches using outcomes such as completion rates, reaction time, and error patterns to better understand participants’ progress (Jaeggi et al., 2011). These data combined with other objective mobile brain and body measures (e.g., EEG, eye tracking, posture) (Greeley et al., 2021) could mark a pivotal step toward establishing it as a rigorous and evidence-based clinical intervention. Critically, more studies are needed to establish the field and to better understand if sand based therapies are effective.

## Conclusion

This review highlights both the lack of research on digital sand therapies in pediatric populations, the lack of scientific rigor in traditional sand-based therapies, as well as the need for standardized outcome measures in sand therapy analyses. The findings demonstrate a significant knowledge gap for the emerging discipline of digital sand therapies. Future research should explore the development of objective, standardized measures for sand therapy analysis through the integration of digital platforms.
