# Supplementary material for: Sand-based therapy in pediatrics: a narrative review of traditional and digital sand therapy
Source: Front Psychol. 2026 Feb 10;17:1692537. doi: 10.3389/fpsyg.2026.1692537 (PMC12929380; doi:10.3389/fpsyg.2026.1692537)
Supplement: Supplementary file 2 [file Table_1.DOCX]

**Methods**

*Search Terms*

Our search terms included: “Digital Gestalt therapy”; “Digital play therapy”; “Digital sand* therapy”; “Online sand*”; “Sand* therapy”; “Technology sand* therapy”; “Telemental health sand* therapy”; “Video game therapy”; “Virtual sand* therapy”

**Data Extraction**

*ChatGPT prompt*

Below is the prompt we used to extract article information using ChatGPT.

“I am trying to extract information from these articles. I will prompt you element by element as to what I'm looking for. Please try to avoid making assumptions or inferences. First, for some of the elements I will ask you to derive the information verbatim to the article, then I will ask for verbatim as well as a summary based on those direct quotes. For every element you find please provide the direct quotes and their corresponding location.

“First please derive the following information from the attached article. For these elements, please provide only the verbatim quotes from the article itself, without inferences: What is the year and title of the article? What is the name and country of the lead author? Provide the DOI of the article. What is the journal it is published in? What type of article is this (research, review, etc)? What are the keywords taken from the abstract (if any)? What are the main questions and/or aims of the study?”

“For the remaining elements, please provide only the verbatim quotes from the article itself, without inferences. Also based on these quotes please provide a summary for each element: How many participants were included in the study (N = ?) What was the age range of participants as well as the mean age? What was the sex of participants (provide a total count per sex of participants)? Describe any other defining characteristics of the participants. What type of study was it (RCTs, double-blind, experimental, cross-sectional, longitudinal, interventional, etc)? What were the measures in the study? Describe the interventions used (please go into details and with specific examples from the text). What were the groups within the study and what were they doing (e.g. what were the control groups doing vs the experimental group)? Briefly describe the sequence of events in the study, please include what measures were used and when). Please provide the primary and secondary outcomes. Please provide the results of this paper. What are the identified gaps in the research? Provide a brief conclusion of the study.”
